# (^18^F)Fluoro-deoxy-D-glucose uptake of knee joints in the aspect of age-related osteoarthritis: a case-control study

**DOI:** 10.1186/1471-2474-14-141

**Published:** 2013-04-22

**Authors:** Young Hoon Hong, Eun Jung Kong

**Affiliations:** 1 Department of Internal Medicine, Yeungnam University School of Medicine, Daegu, South Korea; 2 Department of Nuclear Medicine, Yeungnam University School of Medicine, Daegu, South Korea

**Keywords:** F-FDG, PET, Aging, Osteoarthritis

## Abstract

**Background:**

This study investigated ^18^F-fluorodeoxyglucose (^18^F-FDG) uptake at knee joints for determination of metabolic alteration in association with the advance of age and joint degeneration such as osteoarthritis (OA).

**Methods:**

A total of 166 knees from 83 healthy persons who presented for routine health examination and positron emission tomography-computed tomography (PET/CT) were enrolled in this study. History of knee OA and joint symptoms and signs were reviewed. The maximum standardized uptake values (SUVmax) of cartilage and mean SUV (SUVmean) between the epiphyseal plates of femur and tibia were evaluated at knee joints. Assessment of radiological bony changes was performed using the Kallgren-Lawrence (K/L) grading system with reconstructed CT images of the knee. The joint symptoms and signs were counted and used for diagnosis of clinical and radiological OA of the knee.

**Results:**

The SUVmean of the knee joints showed a remarkable increase with aging in females (r = 0.503, p < 0.01). Remarkable changes of SUVmean were observed with history of OA (p < 0.01). The SUVmean of joint and the intra-articular SUVmax showed higher values in clinical and radiological OA than in normal joints (p < 0.01). Joint-SUVmean showed significant correlation with OA severity graded according to K/L score (p < 0.05). The intra-articular SUVmax showed a significant increase in symptomatic joints, indicating OA in correlation with the joint-SUVmean (p = 0.01).

**Conclusions:**

The increasing ^18^F-FDG uptakes of knee joints showed agreement with aging in females and clinical and radiological knee OA, indicating that the metabolic alterations were consistent with diagnosis and demographic aspect of OA as a surrogate marker for degeneration of the knee in association with aging.

## Background

OA is usually characterized by painful joints, which may accompany subchondral sclerosis, joint space narrowing, and osteophytosis in association with cartilage degeneration. These changes may lead to joint destruction, loss of joint function, and joint failure. Aging is the most important risk factor for primary OA not caused by injury or disease, and almost 80% of people older than 75 years of age present with OA, one of the most commonly occurring diseases in the elderly [1]. However, it remains unclear what occurs in the joints with aging and how aging affects the joints and elicits OA.

Regarding the pathogenesis of OA, it is generally accepted that if physical stress to a joint is sufficient to denature articular cartilage matrix, synthesis of proteoglycans and collagen decreases, making the joint vulnerable to stress [2]. As cartilage wears out, increased stress to the joint may lead to development of a micro-fracture in the subchondral bone. Healing processes following micro-fracture result in bony sclerosis and stiffness, compromising capacity to resolve stress. In a vicious manner, the insulted joints fall into irreversible progression to overt OA [3]. However, metabolic alterations in cartilage may be associated with vulnerability of the joint to stress in primary OA, and certain cellular processes that occur during aging may contribute to development of OA [4].

^18^F-FDG is one of the most popular radiopharmaceuticals used for PET scans and ^18^F-FDG uptake has been reported to be highly sensitive in detection of metabolic alterations in some skeletal disorders [5-8]. Intra-articular SUVmax and joint-SUVmean of ^18^F-FDG on PET/CT were measured and calculated at knees for determination of metabolic changes of the joints in association with aging, grade of bony changes, and presence of clinico-laboratory and radiological knee OA, as defined by the American College of Rheumatology (ACR).

## Methods

This study was designed to evaluate changes of ^18^F-FDG uptake on PET and K/L grades on CT in 166 knee joints from 83 healthy persons (42 males and 41 females). The study was conducted according to the code of ethics of the World Medical Association (Declaration of Helsinki) and all of the procedures followed were approved by the ethical committee of Yeungnam University Medical Center.

### Case procurement

All cases presenting for routine medical examination and whole body fusion PET/CT between 2004 and 2010 were subjected to this study, regardless of joint problems. Any case showing abnormalities on laboratory tests, such as a high erythrocyte sedimentation rate (ESR) and high rheumatoid factor (RF), history of diabetes, malignancy and joint trauma, including surgery, and other metabolic or inflammatory diseases was excluded prior to undergoing PET/CT. Medical history for knee OA was reviewed, and pain, stiffness, crepitus, bony tenderness, and enlargement of each knee were counted from 0 to 5 in total. Body weight, height, and body mass index (BMI) were measured for the standard uptakes of ^18^F-FDG and evaluated in terms of OA as well.

### Integrated ^18^F-FDG PET/CT scan

All patients fasted for at least 6 h prior to undergoing PET/CT (DST or DVCT, GE Medical system, Milwaukee, USA). The serum glucose level was measured to ensure that the result was <200 mg/dL. Sixty to 90 minutes after intravenous injection of 8.14 MBq/kg of ^18^F-FDG, whole-body PET/CT scanning was performed. CT images (120 mAs, 140 kVp, and a section width of 3.75 mm) were acquired without breath-holding instructions. The PET emission scan was obtained immediately following acquisition of CT scan, without change in the patient’s position. Image acquisition for whole body PET/CT was performed separately from the vertex to the mid-thigh in eight to 10 bed positions, and then from mid-thigh to toe in five to six bed positions with an acquisition time of 2.5 to 3 min for each bed position. The attenuation corrected ^18^F-FDG-PET images were reconstructed using the CT data with an ordered-subset expectation maximization algorithm. The attenuation-corrected PET images, CT images, and fused PET/CT images displayed as coronal, sagittal, and trans-axial slices were viewed on a workstation (Advanced workstation 4.4, GE Healthcare). An experienced nuclear medicine physician, who was not aware of the patient’s clinical history or recent radiographic data, reviewed and interpreted the PET/CT images three times. For semi-quantitative analysis, the regions of interest (ROIs) were delineated on trans-axial images, and the maximum and mean standardized uptake values (SUVs) were calculated on the workstation. The SUVs were acquired using the attenuation-corrected images, the amount of injected ^18^F-FDG, body weight of each patient, and cross-calibration factors between ^18^F-FDG-PET and the dose calibrator.

$$\begin{array}{l} \text{SUV}=(\text{decay}-\text{corrected}\;\text{measured}\;\text{activity}\\ \;[\text{mCi}/g]/(\text{administered}\;\text{dose}\;\left[\text{mCi}\right])\\ \;/\text{patient}\;\text{weight}\;[g]) \end{array}$$

**Figure 1:**
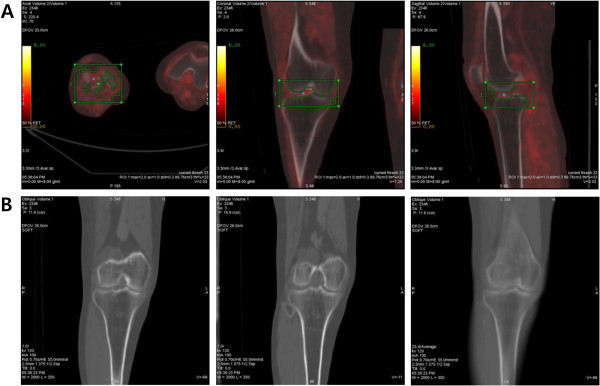
**Joint-SUVmean was measured at the ROIs indicated by the green boxes, which included the minimal area between the epiphyseal plates of the femur and tibia in the trans-axial, coronal, and sagittal planes.** The blue rectangles indicate the points of the highest SUV of intra-articular space, the ROIs for intra-articular SUVmax are precisely delineated by crooked lines within the green box for joint-SUVmean. The point of intra-articular SUVmax was confirmed in trans-axial, coronal, and sagittal slices (**A**). Coronal-reconstruction CT images were arranged in the anterior-posterior view of knees for K/L grading (**B**).

The average value and the highest value in the ROIs were defined as the SUVmean and SUVmax, respectively. Joint-SUVmean and intra-articular SUVmax were adopted in this study because discrimination of joint structures is difficult, and determining which structure is responsible for OA pathology is extremely difficult with PET and CT images. The joint-SUVmean was obtained from the minimal area between epiphyseal plates of femur and tibia, including intra-articular space and subchondral bones of knee joints (Figure 1A, green box). The highest ^18^F-FDG uptake of intra-articular space was determined as intra-articular SUVmax in the zone delineated by crooked lines within the green box for joint-SUVmean, which included cartilage, synoviums, ligaments, menisci, and synovial fluid, except for the epiphyseal parts of femur and tibia, and bony part of patella. Blue rectangles indicate the points of the highest SUV of the intra-articular space (Figure 1A).

### CT image analysis

Non-contrast-enhanced CT images (120 mAs, 140 kVp) were acquired in helical mode with 3.75 mm slice thickness. As for knee joints, the tomographic CT images were reconstructed and assessed according to the procedures outlined by Johnston JD et al. [9]. All reconstructed CT images were evaluated in a blind manner by a PET/CT specialist in axial, coronal, and sagittal views for the presence and degree of joint space narrowing and osteophytes in medial-, lateral femorotibial joints and femoropatellar joint from the knee cap to popliteal fossa at an interval of 1 mm (Figure 1B). Intraobserver reliability was 0.78 with moderate agreement. Subsequently, joint space narrowing and osteophytosis were graded from 0–IV using the Kallgren-Lawrence (K/L) system [10]; no abnormal radiological finding was designated as grade 0; no joint space narrowing but suspicious osteophytes, grade I; minimal joint space narrowing and definite osteophytes, grade II; joint space less than 3 mm, severe intra-articular irregularity, and prominent osteophytes, grade III; no visible joint space and severe sclerosis, grade IV.

### Statistical analysis

Statistical analysis was performed using SPSS program version 12. Cohen’s kappa test was used for determination of intraobserver variation. Independent sample t test, one way ANOVA test, Pearson’s correlation test, and Linear regression analysis were used for parametric analysis and Mann–Whitney test, Kruskal-Wallis test, and Spearman’s test were used for non-parametric analysis, with a confidence interval of 95% and a p value < 0.05.

## Results

### ^18^F-FDG uptake and radiological OA of knee joints

Joint mean and intra-articular SUVmax on PET scan were evaluated according to K/L grade (Figure 1B). Thirty four knee joints were K/L grade 0, 61 were grade I, 38 were grade II, and 33 were grade III, and no cases of grade IV were observed. Mean age of each grade was 46.7±11.3 for grade 0, 55.1±11.5 for grade I, 58.6±10.5 for grade II, and 60.6±8.9 for grade III, respectively; significantly older mean age was observed with a higher grade of bony changes (p < 0.01). Body weight, height, and BMI did not differ among the groups. The mean and max SUV in the group of grade 0 were 1.42 ± 0.36 and 0.85 ± 0.18, respectively; 1.55 ± 0.45 and 0.88 ± 0.20, respectively, in grade I; 1.61 ± 0.58 and 0.96 ± 0.19, respectively, in grade II; 1.70 ± 0.51 and 0.97 ± 0.22, respectively, in grade III. Knees with a higher K/L grade showed higher joint-SUVmean; a significant increase of joint-SUVmean was observed among the groups (p < 0.05), but not for intra-articular SUVmax (Table 1).

**Table 1:** The joint-SUVmean and intra-articular SUVmax of the knees according to K/L grades

		**n**	**Age (yr)**	**BW (kg)**	**HT(cm)**	**BMI (kg/m**^**2**^**)**	**SUVmax**	**SUVmean**
Knee joints	Total	166	55.3±11.7	62.7±11.7	162.9±8.6	23.5±3.2	1.58 ± 0.49	0.91 ± 0.20
	Male	82	55.7 ± 12.1	69.3±11.1**	169.5±6.2**	24.1±3.1**	1.62 ± 0.48	0.94 ± 0.19*
	Female	84	54.9 ± 11.4	56.1±8.2	156.6±5.2	22.9±3.3	1.53 ± 0.49	0.88 ± 0.20
K/L grade	0	34	46.7 ± 11.3	63.9±12.9	163.1±10.3	23.8±3.1	1.42 ± 0.36	0.85 ± 0.18
	I	61	55.1 ± 11.5	62.1±10.4	162.8±7.9	23.3±3.1	1.55 ± 0.45	0.88 ± 0.20
	II	38	58.6 ± 10.5	63.7±14.2	164.1±9.0	23.5±4.1	1.61 ± 0.58	0.96 ± 0.19
	III	33	60.6 ± 8.9	61.3±9.7	161.8±7.4	23.3±2.7	1.70 ± 0.51	0.97 ± 0.22
	IV	0	-	-	-	-	-	-
*p-value*			<0.01**	0.738	0.741	0.916	0.065	0.037*

value = mean ± SD.

BW; body weight, HT; height, BMI; body mass index.

*; p < 0.05, **; p < 0.01.

Knees with K/L grade 0 and I can be classified as non-OA radiographically, and the SUVmean and intra-articular SUVmax of the non-OA group (n = 95, 0.87±0.19 and 1.50±0.42, respectively) were compared with those of the radiological OA (K/L grade II, III, and IV [n = 71], 0.96 ± 0.20 and 1.67 ± 0.55, respectively). The joint-SUVmean and intra-articular SUVmax were higher in the group of radiological OA and the differences were statistically significant (p < 0.01 and p < 0.05, respectively) (Table 2).

**Table 2:** The differences of joint-SUVmean and intra-articular SUVmax by the evidences for knee OA

		**n**	**Age (yr)**	**BW (kg)**	**HT(cm)**	**BMI (kg/m**^**2**^**)**	**SUVmax**	**SUVmean**
Knee + Symptom†	Total	62	57.5 ± 11.8	64.01 ± 1.1	162.6 ± 7.9	24.1 ± 3.3	1.73 ± 0.58**	0.95 ± 0.23
	Male	24	56.0 ± 11.8	70.91 ± 2.1	170.6 ± 5.5	24.2 ± 3.2	1.77 ± 0.60	0.92 ± 0.21
	Female	38	58.5 ± 11.8**	59.7 ± 8.1**	157.6 ± 4.2	24.1 ± 3.4**	1.69 ± 0.57**	0.96 ± 0.24**
Knee without Symptom	Total	104	53.9 ± 11.5	61.8 ± 12.0	163.2 ± 8.9	23.1 ± 3.2	1.49 ± 0.40	0.89 ± 0.19
	Male	58	55.6 ± 12.3	68.7 ± 10.7	169.0 ± 6.4	23.9 ± 3.0	1.56 ± 0.41	0.95 ± 0.19
	Female	46	51.8 ± 10.1	53.2 ± 7.1	155.8 ± 5.8	21.9 ± 2.9	1.40 ± 0.36	0.81 ± 0.15
Knee + OA history		20	64.6 ± 6.8**	63.0 ± 6.1	156.5 ± 2.9*	25.7 ± 2.7**	1.95 ± 0.57**	1.10 ± 0.24**
Clinical§ + laboratory OA		36	61.6 ± 9.9**	64.1 ± 8.1	162.3 ± 7.6	24.4 ± 2.9	1.80 ± 0.54**	0.99 ± 0.23**
Clinical + Radiographic§§ OA		71	59.5 ± 9.8**	64.0 ± 11.1	162.7 ± 7.9	24.1 ± 3.3	1.67 ± 0.55*	0.96 ± 0.20**

value = mean ± SD.

BW; body weight, HT; height, BMI; body mass index.

†; knee joint pain, stiffness, crepitus, bony enlargement and tenderness.

§; ≥ 2 of the symptoms‡.

§§; with definite osteophytes (≥ K/L grade II).

*; p < 0.05, ** ; p < 0.01.

As compared with those of the sex-matched groups without symptom.

As compared with those of the groups without OA history, clinico-laboratory or clinic-radiological OA.

Age and ^18^F-FDG uptake values showed a consistent increase with K/L grade of the knees. In addition, the increase of joint-SUVmean was significantly specific for bony changes and radiological OA of the knee.

### ^18^F-FDG uptake and clinical OA of knee joints

**Figure 2:**
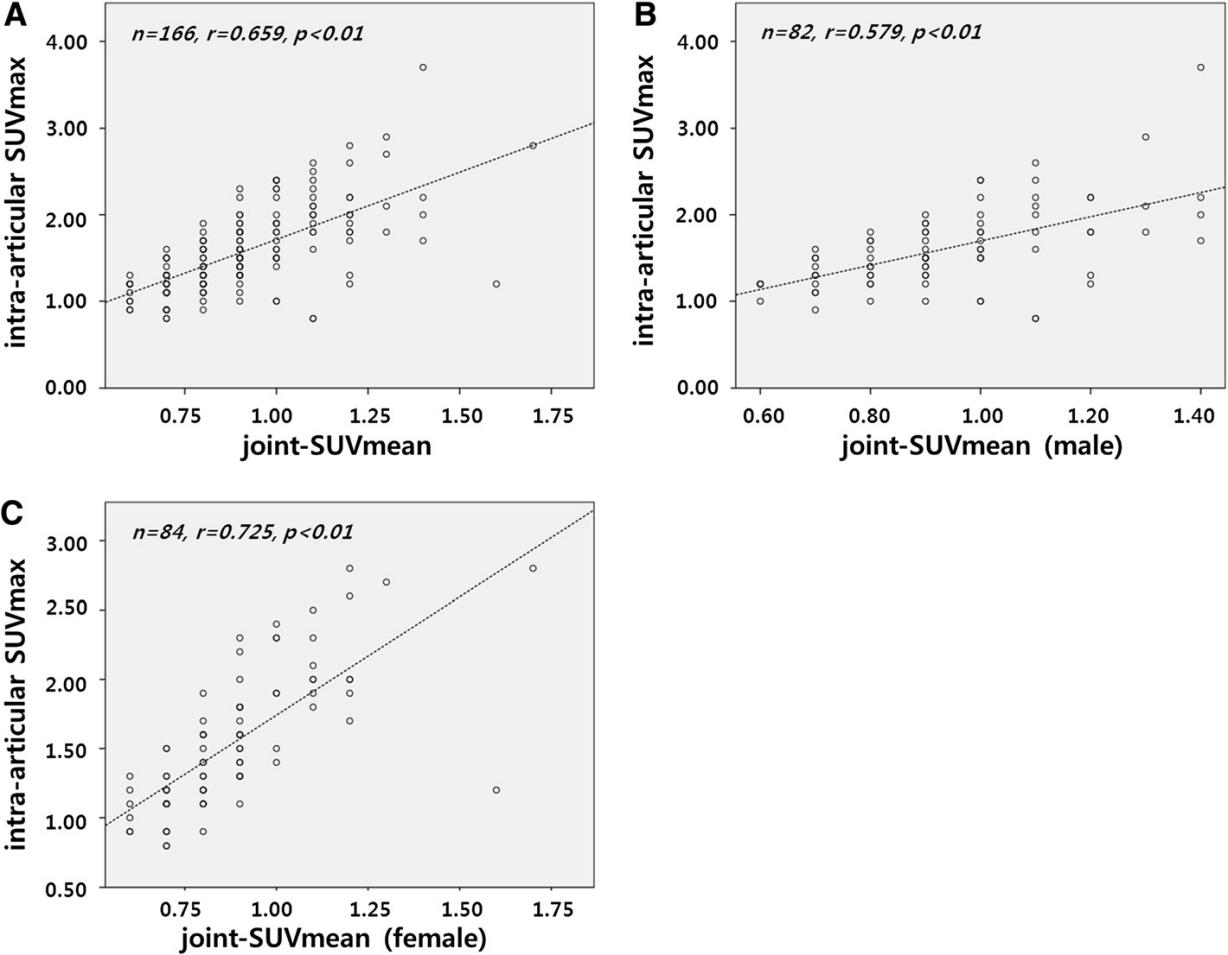
**The difference according to OA symptoms was significant in the intra-articular SUVmax.** The intra-articular SUVmax showed positive correlation with the joint-SUVmean (**A**) in males (**B**) and females (**C**).

The joint-SUVmean and intra-articular SUVmax of knee joints were analyzed according to OA-related symptoms to determine whether the SUVs corresponded to clinical manifestations. OA-related symptoms and signs included knee joint pain, stiffness, crepitus, bony tenderness, and enlargement of the knee according to ACR criteria. They were counted from 0 to 5 in total. No symptoms were observed in 104 knees; 26 knees had one symptom; 12 knees had two symptoms; 12 knees had three symptoms; 10 knees had four symptoms, and two knees had five symptoms. The intra-articular SUVmax with no symptoms was 1.49 ± 0.40; 1.61 ± 0.62 with one symptom; 1.53 ± 0.48 with two symptoms; 1.77 ± 0.47 with three symptoms; 2.06 ± 0.59 with four symptoms; and 2.30 ± 0.42 with five symptoms. Difference among the groups by symptom count for OA was significant for BMI (p < 0.05) and the intra-articular SUVmax (p < 0.01), but not for the joint-SUVmean. However, the intra-articular SUVmax showed strong correlation with the joint-SUVmean (r = 0.659, p < 0.01) (Table 3, Figure 2).

**Table 3:** The differences in joint-SUVmean and intra-articular SUVmax according to OA symptom count of the knees

	**Symptom count of the knee joints**						***p-value***
	**0**	**1**	**2**	**3**	**4**	**5**	
n	104	26	12	12	10	2	
Age (yr)	53.9 ± 11.5	51.9 ± 12.0	58.0 ± 3.6	66.5 ± 4.9	56.4 ± 13.3	81.0 ± 0.0	0.01*
BW (kg)	61.8 ± 12.0	63.9 ± 14.5	66.5 ± 7.9	61.2 ± 8.8	64.2 ± 7.9	67.0 ± 0.0	0.744
HT(cm)	163.2 ± 8.9	163.2 ± 8.5	161.7 ± 8.4	163.8 ± 5.6	162.6 ± 9.1	155.0 ± 0.0	0.836
BMI (kg/m^2^)	23.1 ± 3.2	23.8 ± 3.8	25.4 ± 2.4	22.8 ± 2.8	24.3 ± 2.9	27.9 ± 0.0	0.04*
SUVmax	1.49 ± 0.40	1.61 ± 0.62	1.53 ± 0.48	1.77 ± 0.47	2.06 ± 0.59	2.30 ± 0.42	0.01*
SUVmean	0.89 ± 0.18	0.88 ± 0.20	0.99 ± 0.25	0.90 ± 0.16	1.06 ± 0.29	1.15 ± 0.07	0.051

value = mean ± SD.

BW; body weight, HT; height, BMI; body mass index.

*; p < 0.05.

Because knees with positive RF, ESR higher than 40 mm/h, and palpable warmth were excluded from this study, those with more than two symptoms could be diagnosed as clinical and laboratory knee OA according to ACR criteria. Thirty six knees were included as clinical and laboratory OA; the joint-SUVmean and intra-articular SUVmax of the group were 0.99±0.23 and 1.80±0.54, respectively, which were significantly higher than 0.89±0.19 and 1.51±0.45 of the non-OA group of symptom count 0 and 1 (p < 0.05 and p < 0.01, respectively) (Table 2).

Twenty knees presented with a history of OA, the joint-SUVmean and intra-articular SUVmax were 1.10±0.24 and 1.95±0.57, respectively. The values were significantly higher, compared with 0.91±0.18 and 1.61±0.46, respectively, of the group without history of OA (p < 0.01 for both values) (Table 2).

The results may indicate that the increases of joint-SUVmean and intra-articular SUVmax are consistent with clinical and laboratory knee OA.

### Changes of ^18^F-FDG uptake according to age and gender

**Figure 3:**
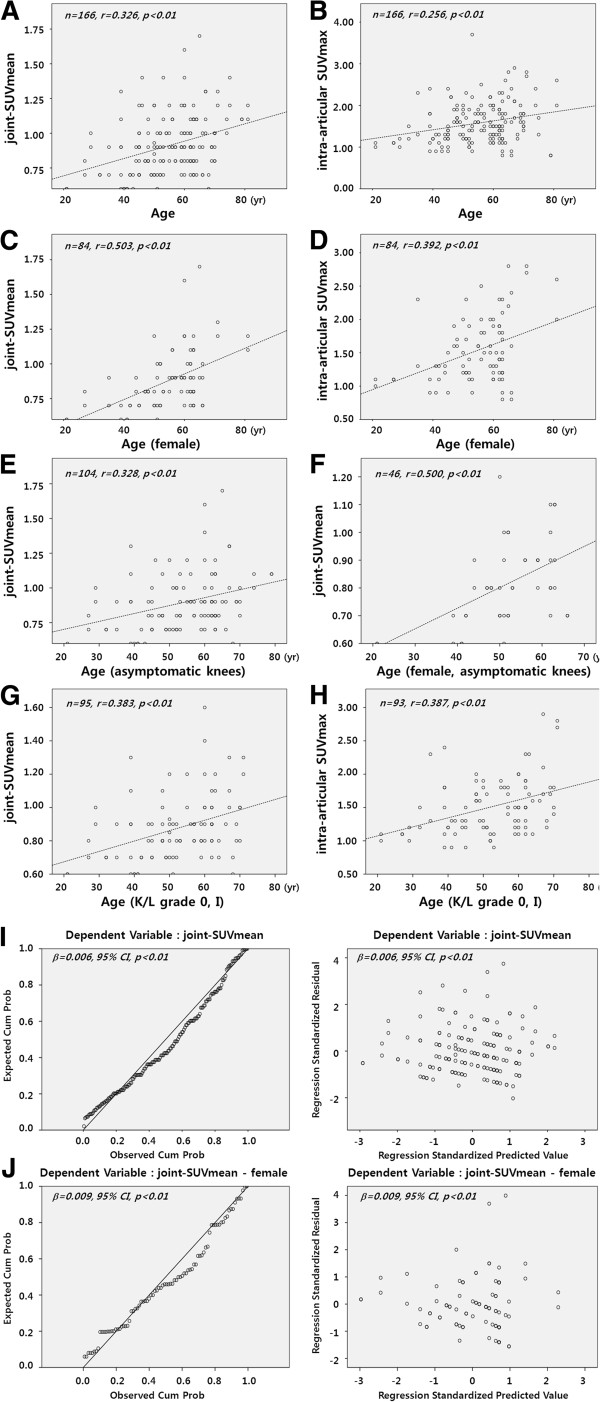
**The SUVs were significantly correlated with age in OA.** With increasing age, the Mean SUV of the knee joint increased independently of the presence of joint problems (**A**, **B**). The joint-SUVmean and intra-articular SUVmax showed significant positive correlation with age (**C**, **D**), even in the group without OA symptoms (**E**, **F**) and without evidence of radiographic OA (**G**, **H**). The SUVs adjusted for age and gender showed remarkable and significant correlations, especially in females (**I**, **J**).

Aging is one of the most important factors in development of OA. As the ACR suggests age above 50 as a criterion for diagnosis of OA, the values for ^18^F-FDG uptake were evaluated according to age. The SUVmean of knee joints showed a weak correlation with the advance of age (r = 0.326, p < 0.01); however, the joint-SUVmean and intra-articular SUVmax in knee joints over the age of 50 (n = 118, 0.94±0.20 and 1.64±0.50, respectively) were significantly higher than those of knee joints under 50 (n = 48, 0.82±0.19 and 1.42±0.41, respectively) (p < 0.01 and p < 0.05, respectively) (Table 4, Figure 3).

**Table 4:** The joint-SUVmean and intra-articular SUVmax of knee joints in the groups over and under the age of 50

			**n**	**BW (kg)**	**HT(cm)**	**BMI (kg/m**^**2**^**)**	**Max SUV**	**SUVmean**
Age (yr)	Total	≥ 50	118	61.9 ± 11.2	161.2 ± 7.8	23.7 ± 3.3	1.64±0.50*	0.94 ± 0.20**
		< 50	48	65.6 ± 12.9	167.3 ± 8.9**	22.9 ± 2.9	1.42 ± 0.41	0.82 ± 0.19
	Male	≥ 50	54	67.6 ± 1.5	167.9 ± 0.8	23.9 ± 0.5	1.68 ± 0.51	0.97 ± 0.18
		< 50	28	72.6 ± 1.9	172.5 ± 1.1	24.3 ± 0.4	1.51 ± 0.43	0.90 ± 0.21
	Female	≥ 50	64	57.0 ± 1.1	155.6 ± 0.5*	23.5 ± 0.4**	1.60 ± 0.50*	0.92 ± 0.21**
		< 50	20	53.4 ± 1.5	160.0 ± 1.6	20.9 ± 0.6	1.31 ± 0.36	0.72 ± 0.09

value = mean ± SD.

BW; body weight, HT; height, BMI; body mass index.

*; p < 0.05, **; p < 0.01.

Body weight and BMI in females with symptomatic knee were significantly higher than in those without symptoms (p < 0.01, respectively). Knees with history of OA had significantly higher BMI and height than those without history of OA (p < 0.01, respectively) (Table 2).

As OA occurs more frequently in females, the intra-articular SUVmax and joint-SUVmean of ^18^F-FDG of 82 knees from 41 males and 84 knees from 42 females were evaluated for determination of the difference according to gender. Mean ages of males and females were 55.7±12.1 and 54.9±11.4, respectively (p = 0.59). The SUVmean in males was 0.94±0.19, significantly higher than 0.88±0.20 of females (p < 0.05). Even higher, the values showed no change by aging in males (r = 0.204, p = 0.06; r = 0.008, p = 0.297, respectively); however, in females, the joint mean and intra-articular SUVmax showed a significant increase in correlation with aging (r = 0.503, p < 0.01; r = 0.392, p < 0.01, respectively). The results may indicate that metabolic alteration represented by changes of ^18^F-FDG uptake is prominent in females, which is consistent with OA development in association with aging and gender.

Adjusted by linear regression analysis for age and gender, the ^18^F-FDG uptake showed an association of each one-year increase in age with a 0.006 increase in joint-SUVmean (β = 0.006, 95% CI, p < 0.01). The βcoefficient of joint-SUVmean adjusted by age and gender in clinico-laboratory and radiological OA was 0.008 (p = 0.002); female 0.009 (p = 0.008) and male 0.006 (p = 0.119), as compared with 0.002 (P = 0.287) in non-OA; female −0.00005 (p = 0.99), male 0.003 (p = 0.271) (Figures 3I,J). Results of the analyses indicated that increased joint-SUVmean in OA was not by aging, and joint-SUVmean in knee OA was related to aging, but not in non-OA, meaning that the significant correlations found between joint-SUVmean and clinic-laboratory and radiological knee OA persist, as compared to non-OA.

## Discussion

ACR criteria for diagnosis of OA require more than three of the clinical-, radiological-, and laboratory criteria. In this study, symptomatic knees that met the ACR requirement for OA showed a significant increase of ^18^F-FDG uptake, and metabolic alteration represented by the change of SUVmean was consistent with a history of knee OA and clinical-, radiological knee OA. The intra-articular SUVmax showed positive correlation with the joint-SUVmean and showed a significant increase in association with joint symptoms indicating OA. The joint-SUVmean showed a significant increase according to the grades of radiological bony changes, and the values were higher in clinical-, laboratory, and radiological OA than those in non-OA.

PET provides molecular imagery of biochemical and metabolic changes and is supposed to overcome the limitations of anatomical imaging studies. Because it is able to illuminate whole body, PET has been widely used for detection and staging of malignancies. For PET imaging, ^18^F-FDG is one of the most commonly used radioisotopes. Because malignant tissues are active in metabolism, increased glucose uptake can be reflected by ^18^F-FDG uptake as a glucose analog [11]. Glucose taken up by cells is metabolized to glucose-6-phosphate and then to fructose-6-phosphate and fructose 1,6-biphosphonate; however, ^18^F-FDG-6-phosphate accumulates in the cell, which creates the images seen on the PET scan [12]. Increased metabolism associated with infection or inflammation can also be detected by PET using ^18^F-FDG; this allows for assessment for inflammatory bone and joint diseases, such as rheumatoid arthritis, psoriatic arthritis, and osteomyelitis [5-8]. OA accompanies metabolic alterations and inflammatory changes; therefore, increasing ^18^F-FDG uptake could be predicted in OA. Wandler et al., who evaluated the pattern and value of ^18^F-FDG uptake in shoulder joints, reported that the uptake pattern was diffuse, including the whole joint in patients with OA [13]. Nakamura et al. reported that ^18^F-FDG uptake was up-regulated in OA and generally accumulated in periarticular lesions [14].

Females are particularly prone to OA, with a risk twice as high as that of males. This has been thought to be associated with estrogen deficiency after menopause, which down-regulates the estrogen receptor on the surface of chondrocytes, resulting in decreased synthesis of proteoglycan [15-17]. In this study, the SUVmean of knee joints was higher in males than in females, however, the changes of the values increased in strong correlation with aging in females, but not in males. This finding might indicate that although metabolic rate is higher in males than in females, the metabolic alteration by aging may be remarkable in females, which is consistent with OA. The differences according to age and gender showed agreement with the higher incidence of OA in aging females than in males.

The glycolysis and pentose phosphate pathways are major pathways of glucose metabolism, and the uronic acid pathway and hexosamine biosynthetic pathways are important for excretion of metabolites as glucuronides and for synthesis of amino-sugars such as glycosaminoglycans (GAGs) and proteoglycans (PGs). In addition to the metabolic pathways, glucose uptake and metabolism can be influenced by the oxygen tension of tissues, thus, decreased tissue perfusion and hypoxia could affect glucose uptake and metabolism by anaerobic glycolysis [18].

Intra-articular and peri-articular structures, including synovium, subchondral bone, ligaments, and synovial fluid, as well as cartilage, may be affected by and generate mechanical loads and soluble mediators directing chondrocytes to activation, cartilage to breakdown, and joints to degeneration [19]. Intra-articular reactions are very important in joint degeneration and pathogenesis of OA; therefore, because discrimination of the respective structures is difficult using PET and CT images, this study adopted intra-articular SUVmax for evaluation of the impact of focal intra-articular reactions on OA. In the current study, the SUVmean of knee joints for global joint metabolism might contain SUVs from peri-articular and intra-articular structures, including subchondral bones, and the intra-articular SUVmax for focal intra-articular reactions irrespective to the structures might involve uptake by synoviums, ligaments, and menisci as well as cartilage.

This study has a specific limitation related to assessment of bony changes of the knee. The K/L grading system of knee joints was originally based on X-ray images, however, in this study, K/L grades were used for the reconstructed CT images of the knee joints [9]. Despite the limitation, the images discriminated bony changes in association with OA and its severity. Even though the increase of joint-SUVmean can be related to direct damage/pathology of articular structures and it is not only related to aging, the change of joint-SUVmean as an indicator for metabolic alteration of knee joints is clearly correlated with presence and severity of primary OA and aging in OA, especially in females.

## Conclusions

As a result of this study, it would be supposed that ^18^F-FDG uptake of PET scan is increased in OA and is consistent with development of OA. The clinical- and radiological evidence for OA and severity of OA consistently showed correlation with the increasing SUVs in association with aging in OA. Early detection for the changes can be a benefit of PET/CT in terms of OA. The ^18^F-FDG uptakes on PET/CT imaging may have capability for early detection of changes to OA beyond the radiological assessment that is critical to preventing progression to irreversible joint failure.

## Abbreviations

F-FDG: ^18^F-fluorodeoxyglucose; OA: Osteoarthritis; PET/CT: Positron emission tomography-computed tomography; SUVmax: Maximum standardized uptake values; SUVmean: Mean SUV; K/L grade: Kallgren-Lawrence grade; ACR: American College of Rheumatology; ESR: Erythrocyte sedimentation rate; RF: Rheumatoid factor; BW: Body weight; HT: Height; BMI: Body mass index; ROIs: Regions of interest; SUVs: Standardized uptake values; GAGs: Glycosaminoglycans; PGs: Proteoglycans.

## Competing interests

The authors declare that they have no competing interests.

## Authors’ contributions

Each author has made substantive intellectual contributions to this study: YHH: participated in collecting data and study design, drafted the manuscript. EJK: participated in collecting data, drafted the manuscript. Both authors read and approved the final manuscript.

## Pre-publication history

The pre-publication history for this paper can be accessed here:

http://www.biomedcentral.com/1471-2474/14/141/prepub
